# Enumerating Microorganism Surrogates for Groundwater Transport Studies Using Solid-Phase Cytometry

**DOI:** 10.1007/s11270-013-1827-3

**Published:** 2014-01-03

**Authors:** Margaret E. Stevenson, A. Paul Blaschke, Sonja Schauer, Matthias Zessner, Regina Sommer, Andreas H. Farnleitner, Alexander K. T. Kirschner

**Affiliations:** 1 Centre for Water Resource Systems, Vienna University of Technology, Karlsplatz 13, 1040 Vienna, Austria; 2 Institute of Hydraulic Engineering and Water Resources Management, Vienna University of Technology, Karlsplatz 13, 1040 Vienna, Austria; 3 Institute for Hygiene and Applied Immunology, Medical University of Vienna, Kinderspitalgasse 15, 1090 Vienna, Austria; 4 Institute for Water Quality, Resource and Waste Management, Vienna University of Technology, Karlsplatz 13, 1040 Vienna, Austria; 5 Institute of Chemical Engineering, Research Group Environmental Microbiology and Molecular Ecology, Vienna University of Technology, Gumpendorfer Straße 1a, 1060 Vienna, Austria; 6 Interuniversity Cooperation Centre Water and Health (ICC), www.waterandhealth.at, Vienna, Austria

**Keywords:** Chem*Scan*™ RDI, Drinking water resources, Groundwater, Microspheres, Solid-phase cytometry, Pathogen surrogates

## Abstract

Investigations on the pollution of groundwater with pathogenic microorganisms, e.g. tracer studies for groundwater transport, are constrained by their potential health risk. Thus, microspheres are often used in groundwater transport studies as non-hazardous surrogates for pathogenic microorganisms. Even though pathogenic microorganisms occur at low concentrations in groundwater, current detection methods of microspheres (spectrofluorimetry, flow cytometry and epifluorescence microscopy) have rather high detection limits and are unable to detect rare events. Solid-phase cytometry (SPC) offers the unique capability of reliably quantifying extremely low concentrations of fluorescently labelled microorganisms or microspheres in natural waters, including groundwater. Until now, microspheres have been used in combination with SPC only for instrument calibration purposes and not for environmental applications. In this study, we explored the limits of the SPC methodology for its applicability to groundwater transport studies. The SPC approach proved to be a highly sensitive and reliable enumeration system for microorganism surrogates down to a minimum size of 0.5 μm, in up to 500 ml of groundwater, and 0.75 μm, in up to 1 ml of turbid surface water. Hence, SPC is proposed to be a useful method for enumerating microspheres for groundwater transport studies in the laboratory, as well as in the field when non-toxic, natural products are used.

**Electronic supplementary material:**

The online version of this article (doi:10.1007/s11270-013-1827-3) contains supplementary material, which is available to authorized users.

## Introduction

Scientists need a dependable method that would make it possible to predict with confidence the setback distance of a drinking water well from a potential point of contamination. Since it is not permissible to perform field tests using pathogenic microorganisms, it is necessary to predict the groundwater transport of hazardous microbes in a different way, using surrogates, such as bacteriophages (Deborde et al. 1999; Schijven et al. 2003) and synthetic microspheres, also known as beads or nanoparticles (Bales et al. 1995; Rudolph et al. 2010). Microspheres can be made from different materials; common materials are iron, silver, latex, polystyrene and silica. For environmental field studies, silica microspheres could be a preferable surrogate due to the presence of silica in natural environments, although a greater variety of polystyrene microspheres are commercially available. In addition to modelling colloid transport in the subsurface, microspheres can also be used to assess the surface–groundwater interaction. For example, fluorescent microspheres have been used to investigate the stream-subsurface exchange of particles in a flume in the laboratory (Arnon et al. 2010), as well as in a natural stream. Groundwater under the direct influence of surface water (referred to as GUDI or GWUDI in North America) is important when determining the vulnerability of a drinking water well to contamination from surface water.

Many different properties have an effect on colloid transport. To name a few, the surface charge of the colloid (depends on pH and ionic strength of the solution), hydrophobicity, size and shape all have an effect on how the colloid particles are transported (Schijven and Hassanizadeh 2000). Thus, for colloid transport experiments, it is more realistic to use surrogates that are approximately the same size and shape as the microorganisms they represent. Although microorganisms are not necessarily spherical, microspheres have been used as a surrogate because they are commercially available and range in size from 0.02 to 10 μm. Viruses, including the bacteriophages sometimes used as surrogates, commonly range in size from 0.02 to 0.30 μm, bacteria from 0.10 to 5 μm and protozoans from 1 to several hundred micrometers. In order to test the efficacy of surrogates as representatives of pathogenic microorganisms, appropriate measuring techniques are mandatory for the comparison. The constraining factor for comparing the transport of microspheres with microorganisms is the detection limit of the measuring method or apparatus. For example, bacteriophages are typically enumerated using the plaque technique, the detection limit being theoretically one plaque forming unit per petri dish, or per millilitre, assuming 1 ml of sample is used for a double-layer plaque assay. Traditionally, microspheres are enumerated using spectrofluorimetry, flow cytometry or epifluorescence microscopy, with detection limits of approximately 1 × 10^8^, 1 × 10^2^ and 5 × 10^2^ particles ml^−1^, respectively, for particles that are 100 to 200 nm in diameter (Pang et al. 2009 and citations therein). The detection limit of the enumeration method is important because the concentration of colloids affects the transport; higher influent concentrations can cause ripening or blocking. Ripening happens when attached cells become favourable attachment sites, enhancing removal, and blocking happens when the attached cells become less favourable attachment sites, resulting in less cell retention. Bradford and Bettahar (2006) found that concentration does have an influence on attachment, but their study was limited to using influent concentrations of 10^5^ to 10^7^ microspheres ml^−1^ due to their enumeration method being spectrofluorimetry.

Solid-phase cytometry (SPC) is an attractive option for enumerating colloids for groundwater transport experiments in the lab and in the field because the detection limit of the method is low (theoretically one particle per scanned filter area; Mignon-Godefroy et al. 1997), the enumeration process is fast and the strain on the technician is minimal, with reproducible results. The SPC detection system consists of a laser that scans the whole area of a filter onto which the target particles have been concentrated and identifies all fluorescent particles (the system aborts if memory capacity is exceeded), which are subsequently discriminated based on settings defined by the user (Mignon-Godefroy et al. 1997). In the past 15 years, several studies have shown that it is possible to detect and enumerate low numbers of labelled microorganisms in sterile and/or environmental waters with SPC (Baudart et al. 2002; Lemarchand et al. 2001; Mignon-Godefroy et al. 1997; Schauer et al. 2012) with one labelled cell detected among 10^7^ to 10^8^ unlabelled (non-target or artificially spiked) cells (Mignon-Godefroy et al. 1997). Reynolds et al. (1999) enumerated *Cryptosporidium parvum* oocysts using SPC (Chem*Scan*™ RDI) in three different river waters, and Hijnen et al. (2005) enumerated *C. parvum* oocysts and *Giardia intestinalis* cysts using SPC for groundwater experiments using columns of sand and alluvial gravel. Fluorescent microspheres are commonly used for calibration of the SPC system, and their detection limit has been tested in sterile water (Lemarchand et al. 2001; Lisle et al. 2004) but, up until now, the limits of detection and quantification of microspheres in environmental water samples (e.g. for groundwater transport studies like column experiments in the laboratory or outdoor in situ experiments) via SPC have not been tested. Environmental waters differ from sterile water because they have differing properties due to suspended particle and microorganism loads. For example, buoyant density affects the different settling rates of beads and bacteria during the filtration process (Lisle et al. 2004), which in turn could affect whether the particles and bacteria settle first or if they settle on top of the beads, subsequently hiding them from laser detection.

The aim of this study was thus to ascertain the limits of detection and quantification of beads via SPC in environmental waters with a special focus on groundwater. This included determining the minimum size of beads that can be detected, the extent of interference from the background matrix and the maximum filterable volume. In addition to groundwater, surface waters with varying background matrices (bacteria and particles) that could potentially influence groundwater resources were investigated to explore the limits of the microsphere/SPC approach.

## Materials and Methods

### Sampling

Water samples representing a wide range of background matrices from sites in eastern Austria (Table 1) were spiked with microspheres and tested with SPC: porous groundwater from the Lobau, a backwater system of the Danube located on the eastern border of the city of Vienna, river water from the Danube and lake water from Neusiedler See and Oberer Stinkersee. Water from the Lobau (National Park Donau-Auen) was sampled from a groundwater well in an alluvial aquifer (AGW1) and from the left bank of the Danube between river kilometre 1,910 and 1,909 (Baart et al. 2010). The sample from the Neusiedler See was taken at a representative station in the centre of the lake (Kirschner et al. 2008), and Oberer Stinkersee water was sampled from the east bank of the saltwater lake (Eiler et al. 2003), which is located within the Neusiedler See - Seewinkel National Park. All samples were taken between January and May, 2011, in clean, sterile 1- or 2-l bottles by first rinsing the bottle with sample water and then taking the sample 30 cm below surface for all except groundwater. Samples were kept cool on ice during transport and were refrigerated at 6 ± 2 °C in the lab until analysis. Additionally, for the determination of the maximum filtration volume possible, karstic groundwater (DKAS1) was obtained from a spring located in the Northern Calcareous Alps (approximately 100 km southwest of Vienna). This particular karstic groundwater system receives a high inflow of surface particles and bacteria during strong rainfall events as well as during snow melt (Farnleitner et al. 2005). All sterile water used for the experiments was autoclaved ion-free reverse osmosis water.

**Table 1:** Measured water quality parameters and total bacteria count

Water Sample	Total Bacteria (cells ml^−1^)	TOC (mg l^−1^)	DOC (mg l^−1^)	TSS (mg l^−1^)	EC (μS cm^−1^)	pH
AGW1^a^	8.00 × 10^4^	2.0	1.7	<5	–	7.5
AGW1^b^	9.24 × 10^4^	1.5	1.4	<5	552	7.3
Danube	1.40 × 10^6^	2.5	1.5	7	429	8.5
DKAS2	1.57 × 10^4^	0.47	0.46	<5	339	7.7
Neusiedler See	9.15 × 10^6^	13.8	12.9	14	1,600	8.7
Oberer Stinkersee	5.85 × 10^7^	67.0	30.9	730	6,400	9.6

Electrical conductivity (EC) for AGW1^a^ was not measured

*TOC* total organic carbon, *DOC* dissolved organic carbon, *TSS* total suspended solids

^a^Sample taken on May, 2011 for SPC tests with beads

^b^Sample taken on October, 2011 for filtration volume tests

### Environmental Parameters

Background bacteria were enumerated in the environmental waters using a Nikon Eclipse 80i epifluorescence microscope and the acridine orange counting method outlined by Kirschner and Velimirov (1997). Water quality measurements were done according to DIN standards (DIN 1987, 1997). Water temperature, electrical conductivity and pH were measured in situ at the time of sample collection using a WTW MultiLine P4 meter (WTW GmbH, Weilheim, Germany).

### Particle Enumeration

#### Particles

Fluoresbrite™ brand of yellow-green fluorescent beads (Polysciences Inc., Warrington, PA) are commonly used for environmental studies (Lisle et al. 2004; Harvey et al. 1989; Knappett et al. 2008) due to their strong fluorescent intensity and emission/excitation spectra being similar to fluorescein (according to the manufacturer), a common dye used for cytometry and epifluorescence application. These carboxylated polystyrene spheres were purchased in various sizes (0.2, 0.5, 0.75 and 1 μm in diameter) and were diluted to desired concentrations using Polysciences Bead Solution (buffer). A ten-fold dilution series was done for each bead size to final stock solutions of 10^4^, 10^3^, 10^2^ and 10^1^ beads per ml. One hundred microlitres of each concentration was mixed with different volumes of each environmental water sample so that the samples contained 10^3^, 10^2^, 10^1^ or 10^0^ beads per sample (not per millilitre).

#### Filterable Volume

Different volumes of each sample were assayed in order to find the limit of background interference for SPC and the optimal sample volume of each water type for practical purposes. The maximum number of allowable particles before the SPC system aborts is defined by the designated memory capacity. This criterion is subject to the total memory capacity of the particular computer being used and therefore, for our experiments, we used the program's default for the number of allowable particles in order to make our results relatively comparable.

#### Solid-Phase Cytometer

The SPC system used for our experiments was the Chem*Scan*™ RDI (AES Chemunex, Ivry sur Seine, France). This system has been used in the past mainly for the detection of bacteria in environmental samples (see citations in Section 1.) and is the only one which is directly connected to an epifluorescence microscope, enabling rapid visual verification of detected signals. The settings of each SPC system vary, and therefore, an optimum balance needs to be found between settings (fluorescence intensity, size etc.) that are broad enough to detect all target particles and narrow enough such that a large number of background particles are not identified after discrimination. The discriminant settings used for the experiments in this paper are listed in the supplemental information (Table S1).

#### Filtration and Mounting

The samples were filtered using a multifold vacuum filtration device (Pall, Port Washington, NY) onto 25-mm black polyester 0.4-μm pore size filters (AES Chemunex). For the 0.2-μm particles, it was necessary to dye 0.02-μm pore size aluminium oxide filters (Anodisc, Whatman, Billerica, MA) with Irgalan black, following the method of Hobbie et al. (1977). The stained filter or black polyester filter was then carefully placed on a support pad (AES Chemunex), which was already saturated with 100 μl of phosphate-buffered saline, prepared on the Chem*Scan*™ RDI sample holder.

#### Validation

Visual confirmation of each event or “hit” (fluorescent particle identified) was done for all samples using a Nikon Eclipse 80i epifluorescence microscope, directly connected to the Chem*Scan*™ RDI system, with a ×100 magnification objective (final magnification: ×1,000). Up to 150 events were validated per filter, and all events were validated when enumeration results were less than 150. At least three replicates were analysed for each concentration and water sample.

### Column Tests

To illustrate the applicability of the SPC method, column tests (similar to set-up described by Jin et al. (2000) for saturated columns) were done using the 0.5-μm beads and influent concentrations of 1.5 × 10^2^, 1.4 × 10^4^ and 1.8 × 10^6^ beads ml^−1^. The columns consisted of 30-cm-long Plexiglas tubes, with an inner diameter of 7 cm, and contained quartz sand (grain size 0.4–0.8 mm) fully saturated with Vienna tap water (pH 8, electrical conductivity 250 μS/cm). The experiments were conducted at a Darcy velocity of 2.7 m/day, pumped upward, and effluent was collected continuously throughout the experiments.

### Statistical analysis

Statistical analysis (Mann–Whitney *U* test, including Bonferroni correction for multiple comparisons) was performed with SPSS Statistics 17.0 software package (Chicago, IL). Results were considered statistically significant when *p* < 0.05 for dual comparisons and < 0.01 for multiple comparisons.

## Results and Discussion

### Particle Size

The smallest particles that could be reliably detected in sterile water were 0.5 μm beads, with minimum intensities of around 200 arbitrary Chem*Scan*™ RDI fluorescent units (FU) per bead. Consequently, the minimum bead size tested in the environmental samples was 0.5 μm, and the results were compared to the enumeration in sterile water for each corresponding concentration. This particle size corresponds well to the size of health related bacteria in environmental waters, like faecal indicator bacteria, for the detection of which a standard filter size of 0.45 μm is used (ISO 2000a). If it were physically possible to manufacture beads with more molecules of fluorochrome per bead, then perhaps particles smaller than 0.5 μm could be detected by SPC (AES Chemunex, personal communication). The minimum particle size detection limit of the Chem*Scan*™ RDI was not tested by Mignon-Godefroy et al. (1997), but the sensitivity of the argon laser was assayed using 1-μm polystyrene beads with 4.5 × 10^4^ molecules of fluorochrome per bead. It was found that beads containing greater than 2,000 molecules of fluorochrome could be detected. Assuming that the number of fluorochromes per bead is directly proportional to the surface area and that fluorochromes are distributed evenly over the surface area, 0.21-μm diameter beads should be detectable with SPC.

### Background Interference

**Fig. 1:**
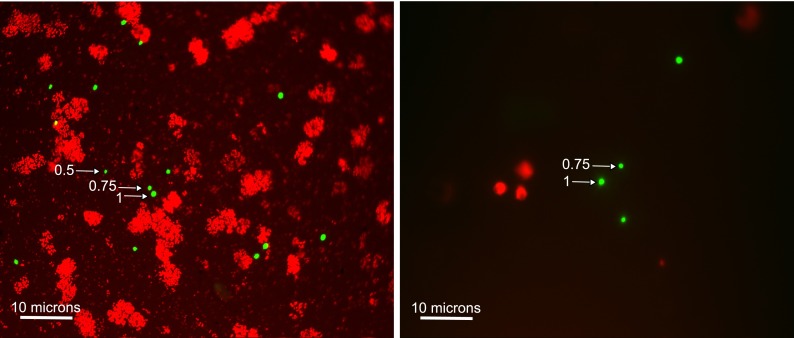
Oberer Stinkersee lake water (*left*) and Danube river water (*right*) exhibit dramatic differences in the concentration of autofluorescent background particles. Background particles are *red* and microspheres (0.5, 0.75 and 1 μm sizes shown) are *green*

Bacterial cells (Lemarchand et al. 2001) or algae present in natural waters may occur in such high numbers that they mask the fluorescence of targeted particles. Furthermore, cyanobacteria or non-target particles retained on the filter cannot only physically cover target cells but can also autofluoresce (Lisle et al. 2004) and could be identified as false positives. These phenomena are illustrated in Fig. 1; the photo on the left shows the high density of background particles (coloured red) present in the Oberer Stinkersee lake water, which partially hid target particles, and the photo on the right shows spherical-shaped background particles (also red) in the Danube river water, which could easily be mistaken for microspheres if it were not for the size and colour settings.

**Fig. 2:**
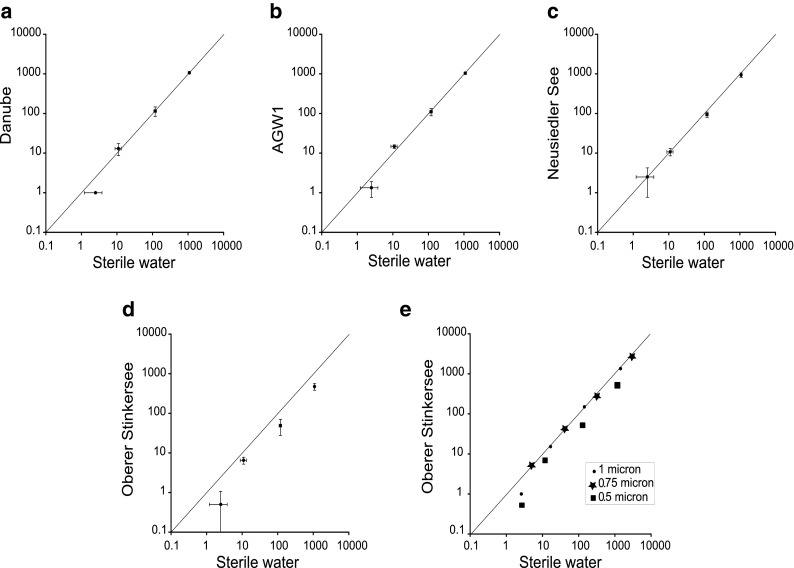
Relationship between bead enumerations (per filter) in sterile water and in Danube river water (**a**), AGW1 groundwater (**b**), Neusiedler See lake water (**c**) and Oberer Stinkersee lake water (**d** and **e**); data points on the 1:1 line represent results that are not negatively impacted by background matrix. *Horizontal* and *vertical bars* (graphs **a** through **d**) represent standard deviations from the mean values (shown) and replicates (*n* = 3–8). Graph **e** shows the relationship for beads of various sizes while graphs **a** through **d** show the results for 0.5 μm beads only

The accuracy of the number of labelled particles detected with SPC can be reduced due to the necessary narrower discrimination settings, causing some targets to be overlooked. However, by optimizing the discrimination settings and visual confirmation, this error can be minimized. The averages of the enumeration results from each environmental sample were plotted against the averages of the results in sterile water for each bead concentration tested. If there was no influence from the background matrix, results should appear on a 1:1 line. To interpret the results shown in Fig. 2, we assume that the bead count in sterile water represents the true concentration and that results appearing on the 1:1 line (shown in Fig. 2) represent high accuracy.

Results close to the 1:1 line are satisfactorily accurate, and results obviously not touching the 1:1 line are considered inaccurate (Fig. 2d, e). It is apparent that accuracy decreases at low concentrations (to be expected) and it was also found that the detection of the 0.5-μm beads in the Oberer Stinkersee water was inaccurate for all concentrations, probably because the high concentration of background particles covered some of the target particles. On average, the percentages of 0.5 μm beads not detectable in the Oberer Stinkersee water were 80, 41, 59 and 56 % for 10^0^, 10^1^, 10^2^ and 10^3^ beads per filter, respectively. Furthermore, at low concentrations, precision decreases on both axes (shown by large standard deviations) due to unavoidable processing error. Table S2, in the supplementary material, shows calculated variation coefficients (indicating precision of collected data) and *p* values from the Mann–Whitney *U* test, which tests for significant differences between the measurement of beads in environmental water and sterile water.

### Detection and Quantification Limits

In practice, there are two kinds of detection limits that have to be considered: method detection limit (MDL) and sample limit of detection (SLOD). MDL is the theoretical detection limit under ideal laboratory conditions for a given method, while SLOD is the estimated detection limit associated with a particular sample (considering sample volume and background matrix) (Domingo et al. 2007). Likewise, there are two kinds of quantification limits: method quantification limit (MQL) and sample limit of quantification (SLOQ). Included in the SLOD and SLOQ are operational variability and intrinsic variability. Operational variability is the uncertainty of the results due to the technical steps of the analytical method, and intrinsic variability is the uncertainty inherent in the random distribution of particles (based on a Poisson distribution) (ISO 2000b). The MDL and MQL of SPC are predefined as one particle per filter, because the SPC system is able to detect and enumerate a single target particle on the whole filter surface (Mignon-Godefroy et al. 1997). SLOD and SLOQ depend on the volume of sample analysed and interference from background matrix and were determined by spiking beads in different environmental waters with different water quality characteristics.

The volume of each sample tested depended on the amount of background material (suspended particles or microorganisms) that caused the SPC to abort, in other words, when the memory capacity is exceeded, as defined by the software, due to too much data. The SPC system aborted when 4 ml of the Danube water was filtered, containing 5.6 × 10^6^ bacterial cells and 28 μg total suspended solids (TSS, calculated from Table 1), which produced excess background fluorescence; therefore, only 3 ml was used for the enumeration tests. One millilitre of Neusiedler See and Oberer Stinkersee water could be analysed successfully, even though the samples contained 9.2 × 10^6^ and 5.9 × 10^7^ cells, as well as 14 and 730 μg TSS, respectively (Table 1). AGW1 water did not cause the SPC system to abort after filtering 50 ml, but the system aborted when 100 ml was used. It was decided that 15 ml of the AGW1 water was the appropriate volume to use for the experiments, considering that this is often the practical volume that is collected per sample during groundwater column experiments in the laboratory (Jin et al. 2000). The sample volume limit of a karstic groundwater sample (DKAS2) was determined for comparison. Five hundred millilitres of the clear karstic water could be filtered and tested with SPC; the SPC system aborted when 1 l was filtered.

The maximum volume of sample water that can be processed for microorganism enumeration is important when determining the SLOD and SLOQ (e.g. 1 particle detected in 1 l represents a 1,000-fold lower detection limit than 1 particle in 1 ml). For our paper, we define the SLOD to be three particles per maximally filterable volume, according to a Poisson distribution of randomly distributed particles (intrinsic variability), assuming a 95 % confidence interval (ISO 2000b). SLOQ is defined as four particles per volume, based on an acceptable relative precision of 50 % (coefficient of variation = 0.50), which seems to be reasonable in microbiology, according to ISO 8199:2005 (ISO 2005). If one requires a higher relative precision of 30 % (CV = 0.30), then SLOQ would be 11 particles per volume (ISO 2000b). Experiments performed in our laboratory (Schauer et al. 2012) showed that as few as 13 microspheres could be reliably quantified with a variation coefficient of 26.5 %, following the protocol outlined in ISO 13843:2000 (ISO 2000b).

From the maximum filterable volumes, the SLOD and SLOQ were calculated (Table 2). Because not more than 3 ml of river water from the Danube could be filtered and processed, the SLOD for river water is one bead per millilitre (three beads per 3 ml; Table 2). One millilitre of Neusiedler See and Oberer Stinkersee was the maximum volume that could be successfully analysed, resulting in an SLOD of three beads per millilitre for highly turbid aquatic environments. Up to 500 ml of DKAS2 water and 50 ml of AGW1 groundwater could be filtered before the SPC system aborted, resulting in SLODs of 0.006 (3 per 500 ml) and 0.06 (3 per 50 ml), respectively (Table 2). The SLODs and SLOQs are improved accordingly as the volume filtered is increased (Table 2); however, 50 ml is a common sample size for microorganism tracer tests in the field (Deborde et al. 1999) and 15 ml is common for laboratory experiments (as mentioned above).

**Table 2:** Limits of detection and quantification for enumeration of fluorescent microspheres of minimum enumerable sizes (0.5 μm for all waters except for Oberer Stinkersee) by solid-phase cytometry

Water Sample	Filterable volume (ml)	MDL (beads filter^−1^)	SLOD (beads ml^−1^)	MQL (beads filter^−1^)	SLOQ (beads ml^−1^)
AGW1	50	1	0.06 (3/50 ml)	1	0.08 (4/50 ml)
Danube	3	1	1 (3/3 ml)	1	1.3 (4/3 ml)
DKAS2	500	1	0.006 (3/500 ml)	1	0.008 (4/500 ml)
Neusiedler See	1	1	3 (3/1 m1)	1	4 (4/1 ml)
Oberer Stinkersee	1	1	3 (3/1 ml)	1	4 (4/1 ml)

A more detailed discussion about limits of detection and quantification can be found in Section 3

*MDL* method detection limit, *SLOD* sample limit of detection (3 is used as the minimum number of particles to be detectable in a water sample according to ISO 13843:2001 [20]), *MQL* method quantification limit, *SLOQ* sample limit of quantification (4 is used as the minimum number of particles to be quantifiable in a water sample according to ISO 8199:2005 [29])

### Column Tests

**Fig. 3:**
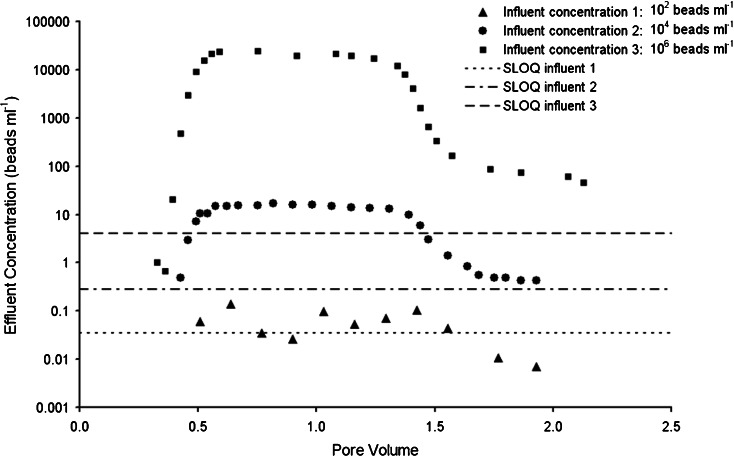
Breakthrough curves and SLOQ values of column tests with influent concentrations of 10^2^, 10^4^ and 10^6^ beads ml^−1^. Data points under the *SLOQ line* are not considered quantifiable. The *x-axis* describes the cumulative volume of effluent water relative to the total water content the column (pore volume)

In order to demonstrate the innovative aspect and practicality of the method, column tests were performed using various influent concentrations, ranging in magnitude from 10^2^ to 10^6^ beads ml^−1^ (Fig. 3). Since traditional enumeration methods, such as spectrofluorimetry, flow cytometry and epifluorescence microscopy, are not able to directly enumerate the range of effluent concentrations observed (10^0^ to 10^4^ beads filter^−1^) without concentration/dilution steps increasing operational variability, such a comparison of concentrations would not have been possible without the use of SPC. For influent concentrations of 10^2^, 10^4^ and 10^6^ beads ml^−1^, sample volumes were 114, 14 and 1 ml and SLOQ values were 0.03, 0.3 and 4, respectively.

## Conclusions

An obvious advantage of using SPC with a surrogate tracer like microspheres is that microspheres are much easier to work with compared to microorganisms; the samples do not have to be kept on ice, groundwater column tests in the lab can be performed at room temperature, no special precautions or training concerning pathogens are required and sterile conditions are not mandatory. If non-toxic, natural materials are used, such as silica, application in field studies is also possible. Another advantage of using SPC with microspheres is that it is both fast and relatively easy compared to cultivation methods. Lastly, the most important advantage of using SPC for groundwater transport experiments is that the detection limit is much lower than that of the traditional methods for enumerating microspheres using spectrofluorimetry, flow cytometry or epifluorescence microscopy. The method allows enumeration of target particles down to a minimum size of 0.5 μm in groundwater, therefore excluding virus-sized particles, for sample volumes of up to 500 ml. In addition, this methodology can also be used for testing the influence of highly turbid surface water (or even wastewater) on a respective groundwater resource, when particles down to a minimum size of 0.75 μm and sample volumes of up to 1 ml are used. We thus propose that SPC is a superior method that can be used to quickly detect and enumerate low numbers of surrogate particles in diverse water matrices.

## Electronic supplementary material

Below is the link to the electronic supplementary material.

Table S1 (DOC 33 kb)

Table S2 (DOC 38 kb)
