# Supplementary material for: Enumerating Microorganism Surrogates for Groundwater Transport Studies Using Solid-Phase Cytometry
Source: Water Air Soil Pollut. 2014 Jan 3;225(2):1827. doi: 10.1007/s11270-013-1827-3 (PMC3928530; doi:10.1007/s11270-013-1827-3)
Supplement: Supplementary file 1 — (DOC 33 kb) [file 11270_2013_1827_MOESM1_ESM.doc]

**Table S1** Discrimant settings used for the Chem*Scan*^TM^ RDI (AES Chemunex, Ivry sur Seine, France). The settings are an edited version of the Vibrio G2000 2Mhz.APP provided by the manufacturer

| **Discriminant Settings** | **Minimum** | **Maximum** |
| --- | --- | --- |
|  |  |  |
| S/P Area Ratio | 0 | 1 |
| T/P Area Ratio | OFF | OFF |
| Single Line Samples | 500 | - |
| Samples | 1 | 250 |
| Lines | 1 | 60 |
| Peak Intensity | 250 | 65535 |
| Peaks Per Line | - | 2 |
| Wiggles Per Line | - | 6 |
| Half Width | - | 15 |
| Specific Intensity (AS) | 3 | - |
| Specific Intensity (HW) | 10 | - |
| 2D Gaussian Fit | - | 1000 |
