# Supplementary material for: Enumerating Microorganism Surrogates for Groundwater Transport Studies Using Solid-Phase Cytometry
Source: Water Air Soil Pollut. 2014 Jan 3;225(2):1827. doi: 10.1007/s11270-013-1827-3 (PMC3928530; doi:10.1007/s11270-013-1827-3)
Supplement: Supplementary file 2 — (DOC 38 kb) [file 11270_2013_1827_MOESM2_ESM.doc]

**Table S2** Variation coefficients and p-values from Mann-Whitney U-test for dilution series with fluorescent beads spiked in different waters. Variation coefficients (ratio of standard deviation to the mean) less than approximately 0.50 are considered to indicate precision and corresponds to the lowest number that can be quantified (= quantification limit), i.e. 4 particles . Calculated p-values from Mann-Whitney U-test show significant differences of measuring beads with SPC in environmental water samples compared to sterile water; multiple comparisons were done for 0.5 µm beads, dual comparisons for 0.75 and 1 µm beads

| **Dilution**  beads filter^-1^ |  | Variation coefficients | | | | Mann-Whitney p-values | | | |
| --- | --- | --- | --- | --- | --- | --- | --- | --- | --- |
|  |  | 10^0^ | 10^1^ | 10^2^ | 10^3^ | 10^0^ | 10^1^ | 10^2^ | 10^3^ |
| **0.5 µm** | Sterile | 0.52 | 0.20 | 0.08 | 0.03 | - | - | - | - |
|  | AGW1 | 0.43 | 0.10 | 0.21 | 0.07 | > 0.1 | > 0.05 | > 0.1 | > 0.1 |
|  | Danube | 0.00 | 0.34 | 0.27 | 0.06 | > 0.1 | > 0.1 | > 0.1 | > 0.1 |
|  | Neusiedler See | 0.69 | 0.21 | 0.15 | 0.12 | > 0.1 | > 0.1 | < 0.05 | > 0.05 |
|  | Oberer Stinkersee | 1.15 | 0.20 | 0.43 | 0.19 | < 0.05 | < 0.05 | < 0.001* | < 0.001* |
| **0.75 µm** | Sterile | 0.20 | 0.08 | 0.09 | 0.04 | - | - | - | - |
|  | Oberer Stinkersee | 0.47 | 0.14 | 0.10 | 0.03 | > 0.1 | > 0.1 | > 0.1 | > 0.1 |
| **1 µm** | Sterile | 0.97 | 0.03 | 0.11 | 0.05 | - | - | - | - |
|  | Oberer Stinkersee | 0.00 | 0.30 | 0.01 | 0.02 | > 0.1 | > 0.1 | > 0.1 | > 0.1 |

* statistically significant (< 0.05 for dual comparisons, < 0.01 for multiple comparisons according to Bonferroni correction)
